# Autophagy in Crotonaldehyde-Induced Endothelial Toxicity

**DOI:** 10.3390/molecules24061137

**Published:** 2019-03-21

**Authors:** Seung Eun Lee, Hye Rim Park, Cheung-Seog Park, Hyun-Jong Ahn, Jeong-Je Cho, Jongsung Lee, Yong Seek Park

**Affiliations:** 1 Department of Microbiology, School of Medicine, Kyung Hee University, Seoul 02447, Korea; eunlee@khu.ac.kr (S.E.L.); hrsabina@khu.ac.kr (H.R.P.); pcs@khu.ac.kr (C.-S.P.); ahnh@khu.ac.kr (H.-J.A.); jjcho@khu.ac.kr (J.-J.C.); 2 Molecular Dermatology Laboratory, Department of Genetic Engineering, Sungkyunkwan University, Seoul 88420, Korea

**Keywords:** crotonaldehyde, cigarette smoke, oxidative stress, autophagy, endothelial cells, cell death, vascular disease

## Abstract

Crotonaldehyde is an extremely toxic α,β-unsaturated aldehyde found in cigarette smoke, and it causes inflammation and vascular dysfunction. Autophagy has been reported to play a key role in the pathogenesis of vascular diseases. However, the precise mechanism underlying the role of acute exposure crotonaldehyde in vascular disease development remains unclear. In the present study, we aimed to investigate the effect of crotonaldehyde-induced autophagy in endothelial cells. Acute exposure to crotonaldehyde decreased cell viability and induced autophagy followed by cell death. In addition, inhibiting the autophagic flux markedly promoted the viability of endothelial cells exposed to high concentrations of crotonaldehyde. Crotonaldehyde activated the AMP-activated protein kinase (AMPK) and p38 mitogen-activated protein kinase (MAPK) pathways, and pretreatment with inhibitors specific to these kinases showed autophagy inhibition and partial improvement in cell viability. These data show that acute exposure to high concentrations of crotonaldehyde induces autophagy-mediated cell death. These results might be helpful to elucidate the mechanisms underlying crotonaldehyde toxicity in the vascular system and contribute to environmental risk assessment.

## 1. Introduction

Cigarette smoke remains an important cause of vascular disease [1]. Exposure to cigarette smoke causes vascular disease via a series of interdependent processes, including augmented oxidative stress and endothelial dysfunction and inflammation [2]. Cigarette smoke is also a co-risk factor for diseases, including diabetes mellitus, hypertension, and haemostasis, further increasing vascular morbidity and mortality [3,4].

Crotonaldehyde is an α,β-unsaturated aldehyde mainly produced during incomplete combustion and is found in relatively large amounts in cigarette smoke (cigarettes contain about 1–53 μg crotonaldehyde/cigarette [5,6]) [7]. Moreover, endogenous lipid peroxidation could result in crotonaldehyde exposures in humans [8]. Acute toxicity of crotonaldehyde is mainly associated with irritation of the eyes, skin, and the respiratory tract [9]. Crotonaldehyde is considered to be an extremely reactive compound and its reactivity corresponds with the mutagenic and cytotoxic effects, which manifest themselves without its prior metabolic activation [10]. Crotonaldehyde induces numerous adverse cellular effects, including inflammatory response and cell death via several mechanisms [11,12,13,14,15].

Autophagy is a complex intracellular process that facilitates protein degradation, cytoplasmic organelle turnover, and recycling of cytoplasmic constituents through lysosome-mediated degradation [16,17]. The incidence of autophagy under physiological conditions, as well as its induction by several stimuli (e.g., nutrient deprivation, ER stress, and oxidative stress) suggests crucial roles for this process in cellular homeostasis [18,19,20]. Recent studies have suggested that autophagy is involved in several fundamental biological processes, including aging, development, cell death, and differentiation [21,22]. Several studies report the regulation and functional importance of autophagy in the pathogenesis of various diseases, including metabolic diseases, kidney diseases, cancer, and vascular diseases [23,24,25]. Numerous studies have also demonstrated the association between endothelial autophagy and vascular risk factors such as cigarette smoke and oxidative stress [26,27]. Nevertheless, the role of crotonaldehyde in autophagy induction and the underlying molecular mechanisms in vascular system remain unclear.

In the present study, we investigated the effects of crotonaldehyde, a major component of cigarette smoke, on induction of autophagy and cell death in human endothelial cells and the underlying molecular mechanisms.

## 2. Results

### 2.1. Induction of Cell Death and Autophagy In Endothelial Cells by Crotonaldehyde

**Figure 1:**
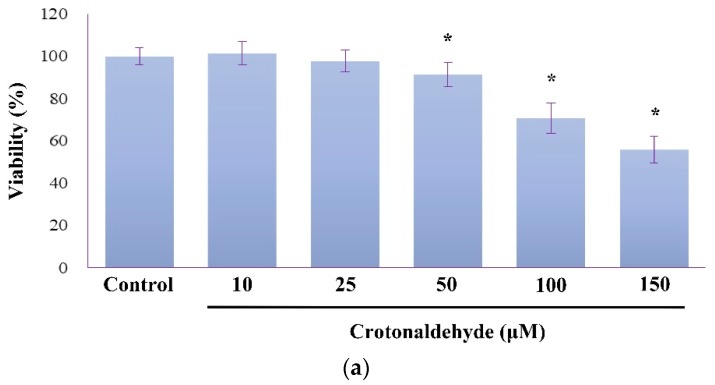

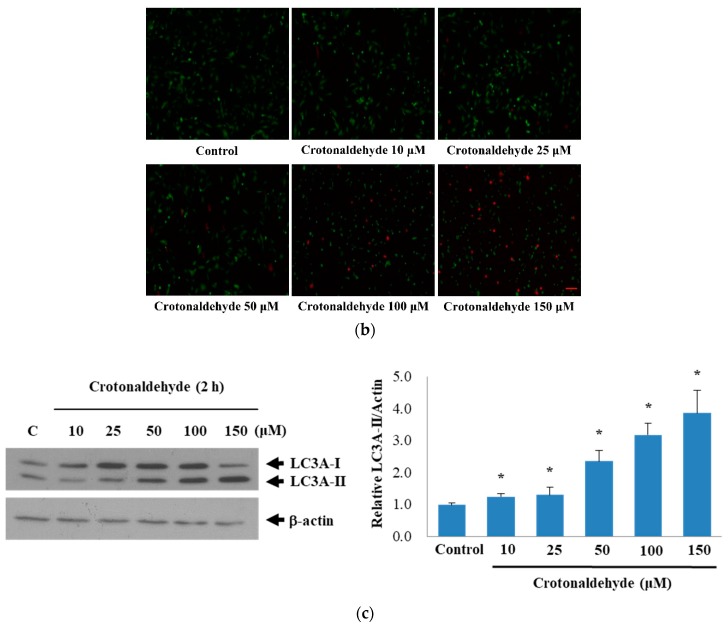
Induction of cytotoxicity and autophagy by crotonaldehyde in endothelial cells (HUVECs). (**a**) MTT assay were used to determine viability of HUVECs exposed to different concentrations of crotonaldehyde (10, 25, 50, 100, or 150 μM) for 2 h. (**b**) Representative images showing cell viability in HUVECs exposed to different concentrations of crotonaldehyde for 2 h. Viability of cells was assayed using the Live/Dead assay kit, live cells exhibit green fluorescence and dead cells exhibit red fluorescence. Scale bars: 100 μm. (**c**) Western blot showing LC3A-II levels in HUVECs exposed to different concentrations of crotonaldehyde for 2 h. The results shown are representative of three independent experiments. Relative densitometric analysis of LC3A-II/Actin expression is shown. Data are expressed as mean ± SD of three independent experiments. * *p* < 0.05 as compared with the control group.

When endothelial cells (HUVECs) were exposed to crotonaldehyde for 2 h, cell viability decreased in a dose-dependent manner, as measured by using the MTT (3-(4,5-dimethylthiazol-2-yl)-2,5-diphenyl tetrazolium) assay (Figure 1a). Results of the Live/Dead assay also showed that crotonaldehyde treatment induced a significant increase in cell death (Figure 1b). Compared to the control cells, cells treated with ≥50 μM crotonaldehyde showed significant decrease in cell viability.

Conversion of microtubule-associated protein 1 light chain 3 (LC3)-I into the autophagosome-specific LC3-II is an autophagy marker that which can be detected using Western blotting. Exposure to crotonaldehyde resulted in considerable increase in the expression of LC3A-II (Figure 1c).

### 2.2. Effect of Crotonaldehyde on Autophagic Flux

Further, we confirmed that the enhanced LC3-II levels observed were due to augmented autophagy rather than an obstruction in any step of autophagy. To verify this, we arrested the LC3-II-mediated autophagosome degradation using the lysosomal protease inhibitor bafilomycin A1 in crotonaldehyde-exposed cells.

**Figure 2:**
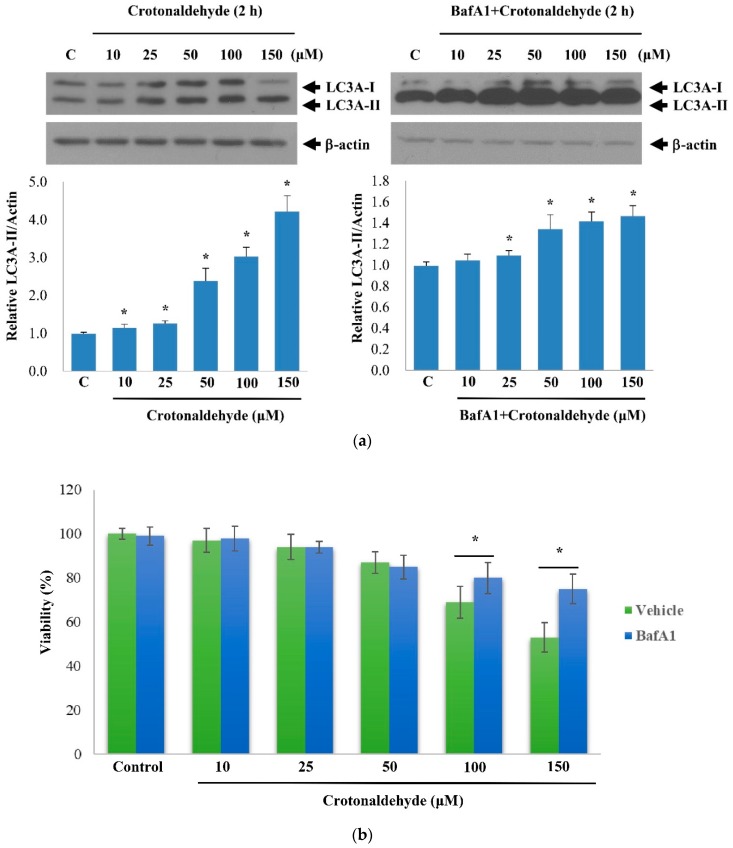
Effect of crotonaldehyde on autophagic flux. (**a**) Western blot showing LC3A-II levels in HUVECs exposed to different concentrations of crotonaldehyde with or without pretreatment of 100 nM bafilomycin A1 (BafA1) for 1 h. Relative densitometric analysis of LC3A-II/Actin expression is shown. Data are expressed as mean ± SD of three independent experiments. * *p* < 0.05 as compared with the control group. (**b**) MTT assays were used to determine viability of HUVECs exposed to different concentrations of crotonaldehyde with or without pretreatment of bafilomycin A1 (BafA1). Data are expressed as mean ± SD of three independent experiments. * *p* < 0.05 as compared with groups treated with corresponding concentrations, without pretreatment of bafilomycin A1.

Compared to the cells treated with crotonaldehyde alone, those exposed to crotonaldehyde in the presence of bafilomycin A1 showed LC3-II lipidation (Figure 2a). These data indicate that crotonaldehyde did not disrupt the autophagic flux but induced autophagy in endothelial cells.

To determine the role of autophagy in the cytotoxic effect produced by crotonaldehyde in endothelial cells, the cytotoxicity of crotonaldehyde was investigated by performing a cell viability assay in the presence of bafilomycin A1. Treatment of cells with crotonaldehyde (150 μM) decreased the cell viability by approximately 60%, whereas combined crotonaldehyde and bafilomycin A1 treatment markedly abolished the cytotoxic effect produced by crotonaldehyde (Figure 2b). This result indicates that autophagy indeed contributed to crotonaldehyde-induced death in endothelial cells.

**Figure 3:**
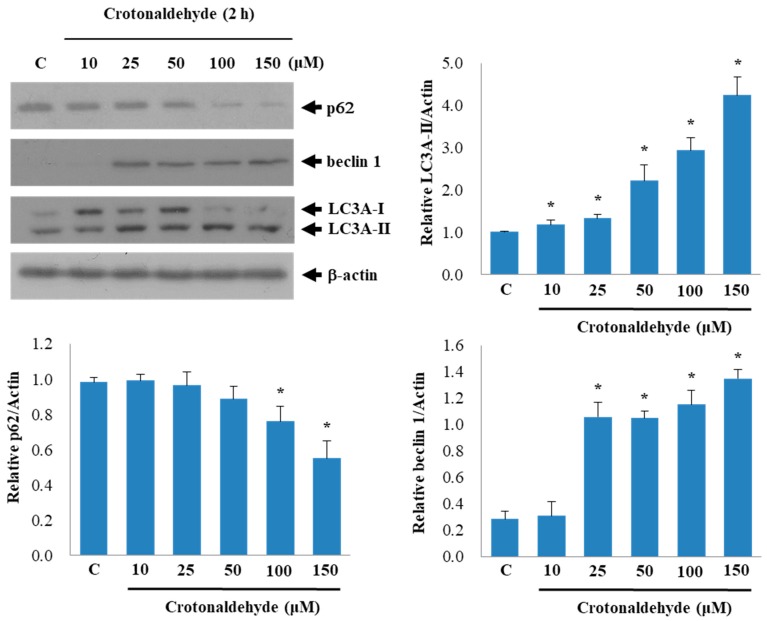
Effects of crotonaldehyde on the expression of autophagic indicators p62 and beclin 1 in HUVECs. Western blotting results showed that the expression of SQSTM1/p62 (p62) was decreased, while beclin 1 and LC3A-II expression was markedly increased in HUVECs exposed to different concentrations of crotonaldehyde. Data shown are representative of three independent experiments. Relative densitometric analysis of LC3A-II, p62, beclin 1 to actin expression are shown. Data are expressed as mean ± SD of three independent experiments. * *p* < 0.05 as compared with the control group.

To further elucidate the effect of crotonaldehyde on autophagy, we measured the expression levels of other autophagy markers, including beclin 1 and sequestosome 1 (SQSTM1)/p62, in crotonaldehyde-treated cells. The expression levels of beclin 1 commonly reflect the level of autophagic activity [28], and the accumulation of p62 implicates impairment of autophagy [29]. A distinct increase was observed in the expression levels of beclin 1 with increasing concentrations of crotonaldehyde; however, p62 expression decreased in the endothelial cells treated with crotonaldehyde, compared with that in the control group (Figure 3). These results suggest that crotonaldehyde augmented autophagy in endothelial cells, thus promoting cell death.

### 2.3. Involvement of AMPK and p38 MAPK in Crotonaldehyde-Induced Autophagy

**Figure 4:**
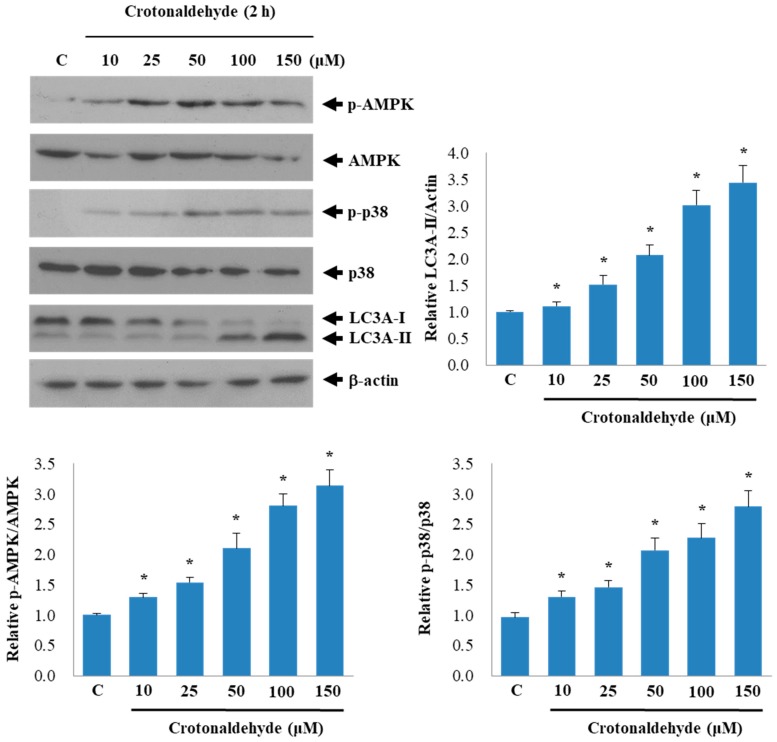
Activation of AMPK and p38 MAPK by crotonaldehyde in HUVECs. Western blot showing the levels of p-AMPK, AMPK, p-p38, p38, and LC3A-II expression in HUVECs exposed to different concentrations of crotonaldehyde. Results shown are representative of three independent experiments. Relative densitometric analysis of LC3A-II/Actin, p-AMPK/AMPK, and p-p38/p38 expression are shown. Data are expressed as mean ± SD of three independent experiments. * *p* < 0.05 as compared with the control group.

**Figure 5:**
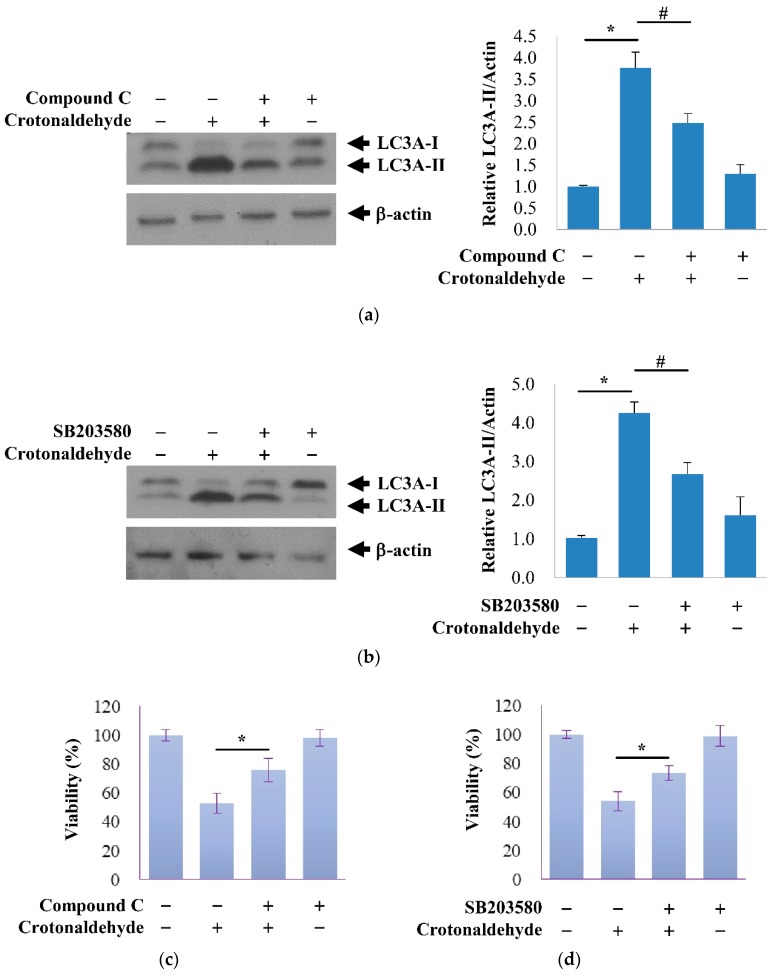

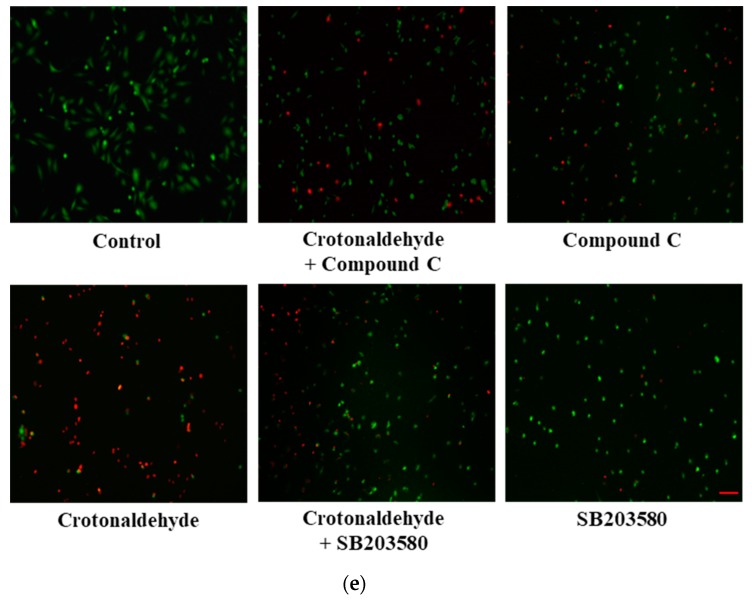
Effects of AMPK and p38 MAPK pathways on crotonaldehyde-induced autophagy and cell death. (**a**) Western blot showing the effect of pretreatment of Compound C, specific antagonist of AMPK, (100 nM, 1 h), on autophagy in HUVECs exposed to 150 μM crotonaldehyde for 2 h. Western blotting results are representative of three independent experiments. Relative densitometric analysis of LC3A-II/Actin expression is shown. Data are expressed as mean ± SD of three independent experiments. * *p* < 0.05 as compared with the control group. # *p* < 0.05 as compared with the corresponding control group. (**c**) MTT assays were used to determine viability of HUVECs exposed to 150 μM crotonaldehyde for 2 h with or without pretreatment with Compound C (100 nM, 1 h). Similar effects of inhibition of p38 MAPK on autophagy and cell death are shown in (**b**,**d**). Data are expressed as mean ± SD of three independent experiments. * *p* < 0.05 as compared with the corresponding control group. (**e**) Viability of cells was assayed using the Live/Dead assay kit, live cells exhibit green fluorescence and dead cells exhibit red fluorescence. Scale bars: 100 μm.

AMP-activated protein kinase (AMPK) is involved in diverse functions such as autophagy, apoptosis, and cell migration [30]. MAPK family members such as extracellular signal-regulated kinase (ERK), c-Jun N-terminal kinase (JNK), and p38 MAPK, have also been reported to be involved in autophagy [31]. Therefore, we investigated whether crotonaldehyde triggers the activation of kinases in association with autophagy. Indeed, crotonaldehyde treatment enhanced the autophagic activity and activation of AMPK and p38 MAPK (Figure 4). To investigate the role of AMPK and p38 MAPK in crotonaldehyde-induced autophagy, cells were pretreated with or without the specific AMPK antagonist Compound C and a p38 MAPK-specific inhibitor SB203580 for 1 h before treatment with crotonaldehyde. Compound C and SB230580 considerably attenuated the crotonaldehyde-induced increase in LC3A-II levels (Figure 5a,b). Corresponding with these findings, the crotonaldehyde-induced increase in cell death was suppressed by Compound C and SB203580 pretreatment, suggesting the involvement of the AMPK and p38 MAPK pathways in the regulation of autophagy-mediated cell death induced by crotonaldehyde exposure (Figure 5c–e).

## 3. Discussion

The World Health Organization (WHO) has predicted that the number of deaths related to cigarette smoking worldwide, by 2030, will increase to over eight million people per year [32]. For several decades, cigarette smoking has been a chief risk factor for cancer and pulmonary diseases [33,34]. Cigarette smoking is also considered as one of the key risk factors for the development of vascular diseases, accounting for 80% elevated risk for coronary artery insufficiency in smokers compared to that in nonsmokers [35]. Clinical and animal studies have clearly revealed that cigarette smoking stimulates endothelial dysfunction and promotes vascular disease [36,37].

Cigarette smoke is a multipotent mixture of several constituents associated with diseases of various organs, and involved in several pathological processes [38]. Cigarette smoke contains α,β-unsaturated aldehydes (acrolein and crotonaldehyde) capable of protein carbonylation, which leads to protein dysfunction, increased oxidative stress, and onset of diseases [39,40]. Crotonaldehyde is an important component of cigarette smoke and a ubiquitously found air pollutant, which has well established toxic effects and could play a crucial role in the etiology of various diseases [41,42]. However, the pathophysiological mechanisms supporting the relationship between acute exposure to highly concentrated crotonaldehyde and vascular disease have yet to be elucidated fully. In this study, we investigated whether crotonaldehyde, a major component of cigarette smoke, modulates autophagy-mediated endothelial cell death.

We focused on the role of autophagy in crotonaldehyde-induced cytotoxicity in HUVECs. In this study, we demonstrated that crotonaldehyde induced acute cytotoxicity in endothelial cells, and increased the expression of autophagy markers. To confirm autophagy induction, we used the autophagy inhibitor bafilomycin A1 (a specific vacuolar type H^+^-ATPase inhibitor), which limits autophagy by inhibiting the fusion of autophagosomes and lysosomes [43]. Combined treatment of crotonaldehyde with bafilomycin A1 elevated expression levels of LC3-II, indicating that crotonaldehyde exposure did not inhibit the autophagic flux. Correspondingly, we found that pretreatment with bafilomycin A1 significantly improved the viability of endothelial cells at or above 100 μM of crotonaldehyde. These results support the association of autophagy with crotonaldehyde-induced cytotoxicity in endothelial cells.

LC3, beclin 1, and p62 are the fundamental autophagy-related proteins involved in an autophagic flux [44]. The conversion of LC3 from LC3-I to LC3-II is regarded as an important step in autophagy. Bcl-2–interacting protein, beclin 1 (Atg6), is an important autophagy marker involved in autophagosome biogenesis, and acts as a chief regulator of autophagy in mammals. Autophagic response of cells via transcriptional activation of the autophagy regulator beclin 1 has been reported [45]. SQSTM1/p62, which is an adaptor protein with numerous binding motifs, interacts with the autophagy machinery as a key adaptor of target cargo. We found that crotonaldehyde induced LC3-II lipidation and increased beclin 1 levels, and decreased SQSTM1/p62 levels, suggesting the activation of autophagy.

Numerous signaling pathways are known to be involved in autophagy induction. AMPK has been reported as a critical regulator of autophagy and was shown to stimulate autophagy [46]. MAPK family members, including JNK, ERK, and p38 have been reported to be involved in autophagy [47]. In this study, we found that activation of AMPK/p38 MAPK contributed to crotonaldehyde-induced autophagy and cytotoxicity, indicating the involvement of these kinases in crotonaldehyde-induced autophagic cell death.

Oxidative stress has been reported as an early inducers of autophagy [48]. Previous studies reported an association between crotonaldehyde and oxidative stress [42,49]. We suggest that crotonaldehyde-induced autophagy occurs downstream of oxidative stress and mediated oxidative stress-induced cell death. Therefore, the autophagy-related molecules may be considered as potential targets to inhibit crotonaldehyde-induced cell damage.

In the present study, we found that crotonaldehyde enhanced autophagic cell death at higher than 100 μM, indicating that acute exposure of highly concentrated crotonaldehyde induced over-stimulated or uncontrolled autophagy. This result may provide a novel insight to help prevent the acute damage caused by potential acute or occupational exposure to crotonaldehyde.

In summary, we demonstrated that exposure to crotonaldehyde, a major component of cigarette smoke, enhances autophagy-mediated cell death in human primary endothelial cells in association with AMPK/p38 MAPK signaling pathways, and the inhibition of autophagy and related kinases partly prevented this cytotoxicity. Our findings may provide novel insights into understanding of the acute vascular damage due to occupational exposure of crotonaldehyde.

## 4. Materials and Methods

### 4.1. Materials

Crotonaldehyde, Compound C, and bafilomycin A1 were obtained from Sigma (St. Louis, MO, USA). SB203580 was purchased from Calbiochem (La Jolla, CA, USA). The following antibodies were used for the present study: p38, phospho-p38, AMPK, phospho-AMPK (Cell Signaling Technology, Beverly, MA, USA), SQSTM1/p62 (Santa Cruz Biotechnology, Santa Cruz, CA, USA), beclin1 (BD Biosciences, San Jose, CA, USA), and β-actin (AbFrontier, Seoul, Korea). All other chemicals and reagents used were of analytical grade.

### 4.2. Cell Culture

Human Umbilical Vein Endothelial Cells (HUVECs), obtained from mixed donors were purchased from Lonza (Basel, Switzerland). Cells were cultured in Endothelial Growth Medium (EGM^™^-2 SingleQuots^™^ Kit: EBM-2 with growth supplements, Lonza). The culture was grown to approximately 80% confluence, maintained using fresh culture medium, and cells were subcultured every 2−3 days. The cells were used within passages 4−9 during these experiments.

### 4.3. Viability Assay

The cytotoxicity of crotonaldehyde was determined using a modified 3-(4,5-dimethylthiazol-2-yl)-2,5-diphenyl tetrazolium (MTT; Sigma, St. Louis, MO, USA) assay, as previously described [49]. We seeded HUVECs in a 96-well plate at 4 × 10^3^ cells/well and incubated these for 24 h in 37 °C with 5% CO_2_. We treated the cells with crotonaldehyde in various concentrations and incubated the cells for a further 2 h. After designated durations of crotonaldehyde exposure, we aspirated the cell culture medium and incubated the cells with 50 μL of medium (containing 12 mM MTT) for 2 h at 37 °C in 5% CO_2_. We then carefully removed the medium and dissolved the reduced formazan crystals in 50 μL DMSO by incubating at 37 °C for 30 min in the dark. We calculated cell viability by measuring the absorbance at 540 nm using a microplate reader (EL 800, Bio-Tek, Winooski, VT, USA) and comparing them with the control cells. All these experiments were performed in triplicate and were repeated independently at least three times.

### 4.4. Live/Dead Assay

The effect of crotonaldehyde on induction of cell death was determined using the Live/Dead assay (Calcein AM & Ethidium Homodimer-1: cat no. L3224, Thermo Fisher Scientific, Waltham, MA, USA). This assay was performed as per the instructions of the manufacturer. Briefly, HUVECs were cultured on 8-well chamber slides at 37 ℃ in a 5% CO_2_ incubator, then, the cells were treated with different concentrations of crotonaldehyde (10, 25, 50, 100, or 150 μM) for 2 h. After designated durations of crotonaldehyde exposure, the cells were incubated with 100 μL of assay reagent containing Calcein AM (2 μM) and Ethidium Homodimer-1 (4 μM) for 1 h at 37 ℃ in 5% CO_2_ and observed under a fluorescence microscope. Live cells were stained with a 2 μM calcein AM solution, while the dead cells were stained with a 4 μM ethidium homodimer solution according to the manufacturer’s instructions. The stained cells (staining is used to determine whether cells are alive or dead), were examined using a fluorescence microscope (Eclipse 50i; Nikon, Tokyo, Japan).

### 4.5. Western Blot Analysis

After pretreatment with the specific AMPK antagonist Compound C (100 nM, for 1 h) and a p38 MAPK-specific inhibitor SB203580 (10 μM, for 1 h) in 150 μM crotonaldehyde-stimulated HUVECs for 2 h, cells were washed with phosphate-buffered saline and treated with RIPA buffer containing 1 mM EDTA, 5 µg/mL aprotinin, 2 µg/mL leupeptin, and 1 mM PMSF, followed by centrifugation at 14,000 × *g* for 15 min. We loaded 20 μg protein from the whole cell lysate to in each lane of a polyacrylamide gel, performed SDS-PAGE, and detected the separated proteins using Western blotting. Horseradish peroxidase-conjugated anti-IgG antibodies (Santa Cruz Biotechnology, Santa Cruz, CA, USA) were used as secondary antibodies to detect the aforementioned protein bands using the enhanced chemiluminescence WESTSAVE-UpTM substrate (AbFrontier, Seoul, Korea).

### 4.6. Statistical Analysis

Statistical significance was estimated using the Student’s t-test, and the results are expressed as mean ± SD.
